# Dissecting gene regulatory networks driving grape berry ripening using comparative systems biology across nonclimacteric fleshy fruits: the NAC-centered hierarchy revisited

**DOI:** 10.1093/hr/uhag176

**Published:** 2026-04-28

**Authors:** Sara Zenoni, José Tomás Matus

**Affiliations:** Department of Biotechnology, University of Verona, 37134 Verona, Italy; Institute for Integrative Systems Biology (I2SysBio), CSIC-Universitat de València, 46980 Paterna, Valencia, Spain

## Abstract

Understanding how fruits ripen holds enormous potential for agro-industrial applications, enabling the development of healthier foods and plant-based medicines. Studying transcription factors (TFs) in nonconventional model species such as grapevine and strawberry remains challenging, yet offers strong translational value, as deciphering the regulatory mechanisms controlling ripening and the accumulation of health-beneficial compounds can support strategies to enhance fruit quality and also mitigate the impacts of climate change. Over the past decade, genome-wide studies in grapevine have applied systems biology approaches to reconstruct gene regulatory networks by integrating temporal transcriptomic and metabolomic data, revealing novel transcriptional relationships underlying berry ripening. Here, we review how these approaches have advanced grapevine research, with particular emphasis on DNA Affinity Purification followed by Sequencing (DAP-seq), a high-throughput method that maps TF binding sites using *in vitro*-expressed TFs and genomic DNA. DAP-seq provides a scalable and cost-effective alternative for TF characterization in grapevine, where stable genetic transformation remains difficult, enabling the rapid interrogation of large numbers of regulators. We further explore how integrating DAP-seq data with temporal transcriptomes from early- and late-ripening cultivars reveals novel regulatory targets and interconnections among known TFs controlling ripening-related processes. Within this framework, we highlight NAC60 as a high-hierarchy regulator of berry ripening, with a potential role in promoting sugar transport and signaling while modulating R2R3-MYB factors controlling specialized metabolite accumulation. Finally, comparative transcriptomic and network analyses in strawberry uncover conserved NAC-regulated ripening modules shared with grapevine, governing key physiological and metabolic transitions, including sugar transport, ABA biosynthesis, chlorophyll degradation, and flavonoid accumulation, supporting conserved regulatory principles across nonclimacteric fleshy fruits.

## Introduction

Wine and table grapes (*Vitis vinifera L.*) are high-value fruit crops due to their diverse range of products, including fresh fruit, wine, juice, and raisins, and their abundance of health-promoting bioactive compounds, which can also be utilized in cosmetic, pharmaceutical, and nutraceutical applications. For example, grape specialized metabolites such as resveratrol result in dietary benefits and play important roles in disease prevention, leading to a longer and healthier life [1]. Grapevines can also be used for research as a model system for woody plants and nonclimacteric fruits [2]. Its economical and scientific importance situates the species in the cornerstone of research and development; however, the grape industry is menaced by the current environmental scenario. Complete yield loss or full plant decay, occurring due to extreme heat, drought, and salinity episodes, are each time more frequent. Even with mild conditions of heat, development is affected, shifting phenological stages such as the onset of ripening and harvest. Ripening, a late phase of berry development, begins at veraison, which coincides with seed maturity and marks the onset of profound metabolic changes that enhance fruit appeal and edibility. This stage is characterized by the synthesis and accumulation of sugars, polyphenols, and volatile compounds, together with the degradation of organic acids and tissue softening, ultimately conferring key quality-related attributes to the berry. These processes are tightly regulated by hormonal signaling and transcription factor activity. As a nonclimacteric fruit, grape ripening is associated with a progressive decline in respiration rate and does not depend on ethylene to complete the process. Instead, many of these changes are governed by abscisic acid (ABA), which begins to accumulate approximately 2 weeks before veraison and reaches peak levels as berries enter the ripening phase [3–5].

Under unfavorable environmental circumstances, fruits do not ripen properly and the accumulation of bioactive compounds is scarce. Understanding the molecular events that control various aspects of ripening (e.g. sugar import) and the accumulation of health-promoting compounds in fruits is essential for developing strategies to mitigate the effects of climate change and to guide future breeding programs, which will likely incorporate gene editing approaches. Advances in next-generation sequencing and computational biology now allow grapevine fruit ripening to be studied from a systems biology perspective, providing a comprehensive view of the interactions among biological entities within the organism rather than focusing on their individual components or functions. Systems biology can now be applied to study any biological process in grapevine like plant development, response to stress, or specialized metabolism. This review provides a foundation for understanding how the integration of multiple omics technologies aids in identifying gene regulatory networks (GRNs) that govern ripening, metabolism, and their underlying molecular processes.

### Transcriptional regulation in the hotspot for the control of grape berry development and metabolism

Gene expression regulation is key to any form of life, as all biological processes, such as tissue differentiation, development and reproduction, and adaptation to the environment, depend on the correct regulation of gene expression. Understanding how gene expression regulation is achieved results in crucial and direct application in agriculture, e.g. through the development of biotechnological strategies focused on improving the quality of crops including the composition of fruits and their timing in ripening and harvest.

Historically, the study of transcriptional regulation has been done following a reductionist approach, where transcription factors (TFs) were studied individually. In grapevine, most cases explore the role of regulators of specialized metabolism (reviewed in Matus, 2016 [6]), driven by the importance of these molecules in grape but also thanks to the simplicity of evaluating their functions when using transient systems for gene characterization (i.e. the appearance of a novel metabolite such as a pigment). These studies involve the characterization of a few isolated transcription factors, by using transient and ectopic stable systems for gene characterization (i.e. the appearance of a novel metabolite). The first identification in transcriptional regulators of secondary metabolism come from the focused study on the control of flavonoid metabolism, an important and widely conserved branch of phenylpropanoid pathway. MYBA1/2 were suggested as transcriptional regulators of anthocyanins, controlling berry color formation [7]. Their functions on activating anthocyanin production were confirmed by transient grapevine cells 5 years later [8]. Later on, several TFs promoting flavonoid (e.g. anthocyanins, proanthocyanins, and flavonols) and stilbenoid biosynthesis were studied by either transient or stable overexpression in heterologous systems such as *Nicotiana tabacum*, *Petunia hybrida*, and *Arabidopsis thaliana* (e.g. see 9–15).

Bogs *et al.* [13] validating the function of individual grapevine genes in their homologous system remains challenging, although transient expression in leaves, hairy roots, and suspension cells has been successfully achieved to confirm the roles of TFs in regulating processes related to ripening or the production of specialized metabolite synthesis (e.g. [16, 17]). ABA has long been recognized, over the past five decades, as a central hormonal trigger of grape berry ripening [18, 19]. In Nicolas *et al*. (2014), the authors identified the ABSCISIC ACID RESPONSE ELEMENT-BINDING FACTOR2 (ABF2) as a critical TF potentially involved in berry ripening. ABF2 regulates ABA-dependent processes, including the expression of genes associated with ripening, such as those linked to sugar accumulation, anthocyanin synthesis, and cell wall modification. Functional studies demonstrated that ABF2 binds to promoter regions of some of the proposed target genes, activating their transcription in response to ABA signaling. This characterization highlighted ABF2’s role as a regulatory hub in coordinating grape berry ripening. However, subsequent studies have yet to delve deeper into its associated regulatory networks or elucidate the precise mechanisms through which ripening is initiated and modulated. Unraveling these details would be crucial to understanding the broader interplay of transcriptional regulators and hormonal cues that drive this complex developmental process.

Unlike model species such as Arabidopsis, maize, or rice, grapevine lacks extensive mutant and transgenic line collections, making functional validation very difficult. The production of transgenic plants requires the regeneration of plants from transformed tissues, which is a long and complex process. Moreover, grapevine has an extended generation time and is considered a recalcitrant crop [20], with a low transformation efficiency (for instance, grapevine may need between 4 and 7 years depending on the cultivar to bear fruit since it is germinated), challenging the use of *in vitro* culture and genetic modification. Nonetheless, some examples of stable transformation in grapevine exist, aiming to overexpress and/or silence the expression of *MYBA* genes. Cutanda-Perez *et al*. ectopically expressed the *VlMYBA1* gene from *V. labruscana* in grapevine hairy root tissue [21]. The transcriptome analysis of these transgenic roots showed that VlMYBA1 regulated a narrow set of genes involved in anthocyanin biosynthesis and allowed to identify novel genes associated with their modification and transport, e.g. the anthocyanin O-methyl transferase (VviAOMT) and the anthocyanin glutathione-S- transferase (VviGST4). Rinaldo *et al*. [22] overexpressed the *V. vinifera* ortholog (*VviMYBA1*) in the white-berried cv. ‘Chardonnay’ resulting in pigmented fruit, and silenced the *VviMYBA1* and *VviMYBA2* genes in the pigmented cv. ‘Shiraz’ producing berries with lightly colored or white phenotypes. The analysis of the transcriptome of berries with altered *VviMYBA* expression demonstrated that *VviMYBA* is a positive regulator of the later stages of anthocyanin synthesis, modification, and transport. Among genes highly correlated with *VviMYBA* expression, the *ANTHOCYANIN 3-O-GLUCOSIDE-O-ACYLTRANSFERASE* (*Vvi3AT*), encoding a BAHD acyltransferase, was found and functionally characterized as a direct MYBA1 target, also demonstrating its ability to produce the common acylated anthocyanins found in grape berries. Later on, Matus *et al*. (2017) overexpressed *VviMYBA1* and the vegetative loci genes *VviMYBA6* and *VviMYBA7* in grape hairy roots and studied their transcriptomes, showing important intra-subgroup differences in the regulation of the anthocyanin branch. These included the exclusive capacity of the berry locus genes (i.e. MYBA1-A2) in regulating the *F3′5′H* gene family, responsible for the synthesis of trihydroxylated anthocyanins (i.e. petunidin, delphinidin, and malvidin) at berry ripening. Due to the extensive number of studies covering the role of these genes, to date, the MYBA TFs are the best characterized members of the anthocyanin-related gene regulatory network.

Thanks to these and many other studies, some initial pieces of the intricate puzzle of gene expression–regulation mechanisms governing secondary metabolism have been positioned. However, despite these advances, we remain far from achieving a detailed and comprehensive understanding of the entire process. This underscores the complexity of these networks and highlights the need for further exploration using genome-wide data and computational tools to elucidate how transcription factors, regulatory pathways, and environmental cues intricately interact to trigger ripening and shape the diversity and abundance of specialized metabolites in the ripening berry.

### Systems biology methods to explore grape gene regulatory networks controlling berry development and metabolism

The grape research and industry communities hold an important reservoir of genetic resources based on wild *Vitis* species and also somatic variants that has led to different cultivars of *V. vinifera* since its domestication. The use of these resources in combination with transcriptomic, metabolomic, QTL mapping, and GWAS studies has helped in the past decades to identify novel GRNs controlling development and metabolism. Recent cases include the identification of orchestrators of mono- and sesqui-terpenoid compounds (aromas; [23]), flavonols (light-protecting and antioxidants; [11]), and regulators of the onset of ripening (also known as veraison; [24]). Despite these examples, the molecular validation of GRNs in grapevine tosses many challenges and difficulties when compared to model species.

In recent years, the rapid expansion of publicly available next-generation sequencing (NGS) data has driven the development of computational tools to predict the functions of uncharacterized TFs, enabling their analysis from a systems-level perspective [25]. Within this paradigm, network theory has emerged as a powerful framework to study transcriptional regulation through GRNs, which model interactions between TFs and their target genes via regulatory binding sites. GRNs are inherently dynamic and context-dependent and can be broadly classified into expression-driven or binding-based networks, depending on the underlying experimental evidence. In grapevine, early GRN studies primarily relied on expression-driven approaches, inferring regulatory relationships from gene coexpression patterns. Early large-scale applications of gene coexpression network (GCN) analysis in grapevine were pioneered by Wong *et al*. [26], who integrated hundreds of publicly available microarray datasets to construct condition-independent and condition-dependent networks covering berry development, stress responses, and specialized metabolism. This work led to the development of VTCdb, an interactive platform that enabled network-based functional inference, module discovery, and prioritization of candidate regulatory genes in grapevine. Building on this foundation, Ghan *et al*. [27] applied RNA-seq-based weighted gene coexpression network analysis (WGCNA) across multiple cultivars and ripening stages to identify conserved transcriptional subnetworks associated with sugar accumulation and ripening. Their study revealed highly connected higher order subnetworks and hub genes linked to chloroplast disassembly, circadian regulation, chromatin remodeling, and senescence-related pathways, highlighting the power of coexpression-based network inference to uncover coordinated regulatory programs during grape berry ripening. In particular, Ghan *et al*. [27] identified multiple TFs within late-ripening subnetworks, including MYB24, whose expression increased with sugar accumulation and clustered with genes involved in senescence-related processes, chromatin remodeling, and metabolic reprogramming. It was not until 6 years later that Zhang *et al*. [23] formally demonstrated its involvement in ripening by integrating multiomics datasets with network analysis.

GCNs have emerged as robust, data-driven *in silico* tools for predicting TF function. In grapevine, multiple studies using transcriptomic datasets from microarray and RNA-seq platforms have demonstrated the value of GCNs as effective proxies for GRNs [28, 29]. However, while these GCNs effectively capture transcriptional coordination, they do not distinguish between direct and indirect regulatory interactions. Beyond computational inference, complementary experimental approaches enable the genome-wide identification of TF binding sites, defining their cistromes and providing direct support for candidate target genes. These binding event-based networks rely on *in vitro* or *in vivo* data and offer a mechanistic layer that complements expression-driven predictions. From all these methods, DAP-seq has been the most explored in grapevine [23, 30–33], as it is highly reproducible and offers better resolution in binding profiles (i.e. peaks) when compared to Chromatin Inmunoprecipitation followed by sequencing (ChIP-seq) [34, 35], while also overcoming the sensitivity and specificity requirements of specific antibodies for ChIP-seq. DAP-seq has been successfully adapted to fruit crops, with specific and dedicated methodologies now established [36].

DAP-seq, developed by O’Malley *et al*. [37], is an *in vitro* approach for genome-wide identification of TF binding sites. Genomic DNA is fragmented (~200 bp), and incubated with *in vitro*-expressed, affinity-tagged TFs immobilized on beads. DNA fragments containing TF binding sites are retained, eluted, PCR-amplified, and sequenced. Because DAP-seq does not require TF-specific antibodies, it is a scalable and cost-effective method suitable for high-throughput analysis of large TF collections. Despite lacking chromatin context and cofactor interactions, DAP-seq recovers a high proportion of ChIP-seq peaks [38]. Recent extensions such as double DAP-seq enable the analysis of TFs acting as complexes [34], further increasing its applicability, particularly in nonmodel plant species where transgenic approaches are challenging.

While DAP-seq identifies many potential TF binding sites, it does not confirm whether these sites lead to activation or repression. Often, the regulatory role of a TF (as an activator or repressor) depends on associated cofactors, which function within specific biological contexts. Furthermore, the combination of DAP-seq and other datasets offers a consistent approach for the study of GRNs in grapevine. For example, the overlap between aggregated gene-centered networks and DAP-seq [23] or DAP-seq and transcriptome responses to transient TF overexpression (e.g. Orduña *et al*. [39]) have resulted in a reliable set of high confidence targets (HCTs), some of which have been validated using dual-luciferase assays.

More recently, chromatin accessibility profiling has emerged as a powerful complementary layer to refine GRN inference in grapevine by identifying regulatory regions that are permissive to TF binding in specific physiological contexts. Ren *et al*. [40] combined Assay for Transposase-Accessible Chromatin using sequencing (ATAC-seq) and RNA-seq to map condition-specific open chromatin regions in the wild *V. amurensis* during cold stress, enabling the identification of stress-responsive TFs and their associated regulatory modules. By integrating chromatin accessibility with coexpression networks and functional assays, this study demonstrated how TF-centered networks can be resolved beyond correlation to include regulatory potential at accessible *cis*-regulatory elements [40]. Extending this framework to metabolic regulation during fruit development, Yang *et al*. [41] integrated ATAC-seq and RNA-seq with transient expression assays to identify TPS12 and HMGR2 as key targets of light-responsive regulation in grape berry wax biosynthesis. Their study further demonstrated direct binding of TFs HY5 HOMOLOG (HYH) and GATA24 to accessible chromatin regions in the promoters of these genes, linking chromatin accessibility, TF binding, and metabolic output within a coherent gene regulatory network.

### Integration of transcriptome and cistrome methods for dissecting berry ripening GRNs

Grape berry ripening is a highly coordinated process regulated by complex gene networks and TFs. The first studies using network theory to explain key TFs and hallmarks of grape berry ripening integrated large transcriptomic datasets from temporal series across different cultivars and seasons, spanning both pre- and postveraison berry stages. Palumbo *et al*. [42] introduced an integrative network-based approach to analyze gene expression in grapevine. They identified a small group of genes, called ‘switch genes’, categorized as ‘fight-club hubs’ showing strong negative correlation with network neighbors, and proposed them as master regulators during the transition from immature to mature growth. Switch genes were found to be highly expressed in mature tissues but downregulated in ripening-deficient mutants, emphasizing their critical role in coordinating fruit maturation processes. Building on this, Fasoli *et al*. [43] conducted a comprehensive transcriptomic and metabolomic analysis of grapevine berry development. They mapped coordinated waves of gene expression from early development to late ripening, focusing on two rapid transcriptomic transitions at veraison, marking the onset of ripening. Their work confirmed the TFs previously identified by Palumbo *et al*. [42] while also identifying new TFs potentially driving hierarchical gene activation cascades. Together, these studies enhanced our understanding of the genetic and molecular mechanisms regulating ripening and, most importantly, established a list of TFs for later characterization using systems biology approaches. Many of these TFs belong to the R2R3-MYB and NAC families, composed of 134 and 74 members [44, 45], respectively. Some of these have been characterized in grape and are associated to the control of fruit ripening and fruit-specialized metabolism. Here we review their expanded roles thanks to the use of GCNs, cistromes (DAP-seq), and transcriptomics.

### Conserved roles of S2- and S6-R2R3 MYB genes in regulating the shikimate and early phenylpropanoid pathways, despite their regulation of different end-branch phenylpropanoids.

Orduña *et al*. [39] recently expanded the role of TFs from Subgroup 2 of the R2R3-MYB family. The members of this subgroup (e.g. MYB14 and MYB15) play a key role in regulating the biosynthesis of resveratrol and other stilbenoid derivatives [15], by activating stilbene synthase (*STS*) genes. By combining GCNs, DAP-seq data, and TF-transient overexpression followed by microarrays, the authors identified additional targets belonging to earlier pathways; i.e. the shikimate and early phenylpropanoid pathways, including *DAHP synthase*, *Shikimate kinase*, *EPSP synthase*, *Chorismate synthase*, *PAL*, *C4H*, and *4CH* genes. These observations allowed them to suggest that these MYB regulators increased the flux into the production of these compounds. Later on, Orduña *et al*. (2023) showed that the same methods (this time using TF–hairy root stable overexpression followed by transcriptomics) was sufficient to corroborate *MYBPA1* as a direct regulator of proanthocyanidin (PA) biosynthesis. Notably, this control seems to start at the shikimate pathway (i.e. a primary metabolic pathway responsible for the production of phenylalanine) as at least one isoform from each step of the pathway was bound by this TF. MYBPA1 also controlled early phenylpropanoid pathway genes responsible for the accumulation of p-coumaroyl-CoA (i.e. PAL, C4H, and 4CL), one of the substrates of chalcone synthases (also targets of MYBPA1), required to produce flavonoids. The robustness of *MYBPA1* target identification was supported by the fact that all *MYBPA1*-characterized targets related to the proanthocyanidin biosynthesis [13, 14, 46] were bound and coexpressed with *MYBPA1*. DAP-seq results showed a binding peak in the promoter region of the *MYBPA1* gene, suggesting a *MYBPA1* autoregulatory loop. Additional targets included the PA-biosynthesis regulators MYBPA2 and MYBPAR, and *MYC1* that acts as a flavonoid bHLH coregulator.

MYB14/MYB15 and MYBPA1, despite belonging to distant phylogenetic subgroups within the R2R3-MYB family, share a very similar binding motif, the AC-element. This similarity may explain why *MYBPA1* targets the same genes of the shikimate and early phenylpropanoid pathway as *MYB14*/15. On the other hand, small differences in the binding motif and bigger divergence in the C-terminal end of *MYBPA1* and *MYB14*/15 may explain the specificity for the regulation of proanthocyanidin and stilbenoid biosynthesis, respectively.

### A single S9-R2R3 MYB gene controls the accumulation of terpenes at late berry ripening

By integrating multiomics data within a network-based framework, recent studies have moved beyond candidate gene identification to achieve comprehensive functional characterization of individual regulators of fruit ripening. Zhang *et al*. [23] provided a comprehensive functional characterization of MYB24, establishing it as a late-ripening transcriptional hub that integrates developmental and environmental cues to coordinate terpene biosynthesis, carotenoid metabolism, and flavonol-mediated photoprotection, thereby revealing a central role in light-responsive metabolic reprogramming during late grape berry ripening. By combining GCNs, DAP-seq, transcriptomics, metabolomics, and functional assays, the authors exemplified how multiomics integration can dissect complex berry ripening gene regulatory networks and uncover TFs that bridge fruit quality traits, such as aroma, with stress-adaptive metabolic responses.

*MYB24* was initially identified through transcriptome analyses of variegated berries, where anthocyanin-depleted skin sectors exhibited a strong induction of MYB24 concomitant with increased expression of genes associated with isoprenoid metabolism, photosynthesis, and light stress responses. GCN analyses positioned MYB24 within a tightly connected module enriched in terpene synthase (*TPS*) genes, particularly *TPS35*, *TPS09*, *TPS10*, and *TPS14*, whose expressions also peak at late ripening stages and correlate with monoterpene accumulation. The integration of DAP-seq, coexpression networks, and multicondition transcriptomics enabled the identification of a robust set of MYB24 HCTs. DAP-seq revealed that MYB24 binds preferentially to AC element-containing promoter regions of 22 *TPS* genes, with a strong enrichment near transcription start sites, supporting a direct regulatory role. Functional validation using transient dual-luciferase assays demonstrated that MYB24 activates the promoters of *TPS35* and *TPS09*, in a mechanism dependent on interaction with the bHLH cofactor MYC2, mirroring the regulatory logic observed for its Arabidopsis homologs [47]. Consistently, monoterpene profiling confirmed increased accumulation of compounds such as geraniol, nerol, citronellol, and α-terpineol in tissues where *MYB24* expression is elevated, particularly at late ripening stages and under conditions of enhanced radiation exposure.

Beyond aroma-related terpenes, MYB24 was shown to integrate isoprenoid and carotenoid metabolism into a broader light-adaptive response. DAP-seq and expression analyses identified several carotenoid pathway genes among MYB24-bound targets, including *CRTISO2*, *Z-ISO*, *LCYE*, *ZEP1*, and *NCED6*, many of which were transcriptionally induced in anthocyanin-depleted berry skins. Transactivation assays further confirmed MYB24-dependent activation of the *CRTISO2* promoter, supporting a direct contribution of MYB24 in promoting carotenoid accumulation or remodeling during late ripening, particularly under high-light conditions.

A key conceptual advance of this work is the positioning of MYB24 as a light-responsive regulator that compensates for reduced anthocyanin-mediated photoprotection. *MYB24* expression is strongly induced by UV-B and high visible light and is negatively correlated with anthocyanin content, consistent with a light dosage-dependent response. Importantly, MYB24 directly binds to and activates the promoter of *HYH*, a central regulator of early light responses previously characterized in grapevine [48]. Through HYH, MYB24 indirectly promotes the activation of the flavonol regulator MYBF1 and downstream flavonol biosynthetic genes such as *FLS1* and *GT5*/*GT6*, leading to increased accumulation of UV-shielding flavonols. This MYB24–HYH–MYBF1 regulatory axis provided a mechanistic link between light perception, transcriptional control, and specialized metabolite accumulation in ripening berry skins.

### Regulation of ripening, overripening, and associated processes by NAC genes

By combining GCNs, DAP-seq, and transcriptomic data obtained from grapevine leaves stably overexpressing NAC60, D’Incà *et al*. [32] demonstrated that this transcription factor exerts a broad regulatory role in secondary metabolism, programmed cell death, and organ degreening. NAC60 had previously been identified as a switch gene, i.e. a putative master regulator of the vegetative-to-mature phase transition in grapevine organs [42, 49]. Now, integrative analyses positioned NAC60 at the core of a hierarchical gene regulatory network controlling the onset of ripening-associated processes. Promoter activation assays revealed that NAC60 directly induces chlorophyll degradation and anthocyanin accumulation through transcriptional activation of *STAY-GREEN PROTEIN 1* (*SGR1*) and *MYBA1*, respectively. Importantly, *MYBA1* activation was shown to depend on the formation of a regulatory complex between NAC60 and NAC03, highlighting combinatorial control as a defining feature of NAC-mediated regulation. In addition, MYB14, a key regulator of stilbene biosynthesis [15, 39], was identified among NAC60-bound genes, and dual-luciferase assays confirmed its direct transcriptional activation by NAC60, thus also linking NAC60 to the activation of stress-related specialized metabolism.

The integration of DAP-seq data mapped onto the color locus haplotypes provided further mechanistic insight into NAC-mediated regulation. By aligning DAP-seq reads from NAC60 and NAC03 libraries onto both the white (*GRET1*-containing) and red (wild-type) alleles of the *MYBA1* locus, D’Incà *et al*. [32] identified highly proximal binding events for both TFs in both haplotypes, as well as an additional upstream NAC60 binding site preceding the *GRET1* insertion in the white allele. The spatial proximity of these binding events supports a cooperative interaction between NAC60 and NAC03 at the MYBA1 promoter. Notably, the NAC60 binding motif identified at this locus (CACGTAAC) diverged from the dominant motif found in the TOP600 genome-wide peaks (AATGGGAC), suggesting locus-specific binding configurations that may contribute to regulatory specificity during ripening. Two nonexclusive explanations could account for this: (i) NAC60 may exhibit flexible/dual binding preferences depending on local sequence context and peak-calling stringency, or (ii) NAC60 binding at *MYBA1* may be stabilized by partners TFs (e.g. NAC03), effectively shifting the enriched composite motif at that locus. This interpretation is consistent with the experimentally supported combinatorial activation of *MYBA1* by NAC60 together with NAC03.

Extending the role of NAC TFs into late and postripening stages, Foresti *et al*. [31] functionally characterized NAC61, an NAC family member whose expression sharply increases during postharvest berry dehydration and closely correlates with sugar concentration. Through transient overexpression in *N. benthamiana* and grapevine leaves, combined with transcriptomic profiling, DAP-seq, and GCN analyses, NAC61 was shown to regulate gene sets associated with secondary metabolism, osmotic and oxidative stress responses, and biotic stress signaling, processes that typify late ripening and overripening stages. High-confidence NAC61 targets included *STS36*, the stilbene regulators *MYB14*, *WRKY03*, and *WRKY43*, as well as the additional R2R3-MYB genes *MYB163* and *MYB164*, previously associated with ABA response and cell wall degradation, and phenylpropanoid metabolism, respectively (see Supplementary Tables S6 and S7 in Cavallini et al. (2016). Beyond specialized metabolism, NAC61 directly activated genes involved in dehydration and stress adaptation, including the dehydrin gene *DHN1b*, which is strongly induced in withering berries and under water deficit, and *WRKY52*, coding for a known susceptibility factor for *Botrytis cinerea* [50]. These direct regulatory relationships, validated by dual-luciferase assays, established NAC61 as a transcriptional hub coordinating stilbenoid biosynthesis with osmotic, oxidative, and biotic stress responses characteristic of late and postripening grape berries. Also importantly, Foresti *et al*. [31] revealed that NAC61 operates within a higher order NAC regulatory network. NAC60 not only transcriptionally activates NAC61 but also physically interacts with it, forming a NAC60–NAC61 heterodimer that enhance the activation of shared targets such as *MYB14* and reinforce NAC61 self-activation. Together with previously reported NAC60 homodimers and NAC60–NAC03 interactions [32], these findings support a model in which ripening and overripening are governed by a dynamic NAC–NAC interaction network, enabling fine-tuned control of developmental progression, stress adaptation, and secondary metabolism. Current evidence supports both homo- and heterodimerization among NAC TFs [51, 52] while a few additional studies support tetramerization [53]; thus, the predicted stoichiometry of the network likely involves dynamic partner switching across developmental contexts, rather than a single obligate dimer.

Table 1 reports the validated NAC–NAC physical interactions achieved to date by Bimolecular Fluorescence Complementation (BiFC) assay in grape protoplasts and their synergistic interactions in the activation of common target genes revealed by combined dual-luciferase assay.

**Table 1:** Validated NAC–NAC interactions in berry ripening-related NAC TFs

**TF pair**	**Evidence type**	**Biological context**	**Functional consequence**	**Reference**
NAC60–NAC60	Western blot analysis, BiFC	Grapevine berry Grapevine callus protoplasts	Achievement of regulatory activity of NAC60	[32]
NAC60–NAC03	BiFC, Combined dual-luciferase assay	Grapevine callus protoplasts	Synergistic induction of *MYBA1*	[32]
NAC60–NAC33	BiFC	Grapevine callus protoplasts		[32]
NAC03–NAC33	BiFC, Combined dual-luciferase assay	Grapevine callus protoplasts	Synergistic induction of Stay green 1 (SGR1) expression	[32]
NAC60–NAC61	BiFC, Combined dual-luciferase assay	Grapevine callus protoplasts	Synergistic induction of *NAC61* and *MYB14* expression	[31]

**Figure 1:**
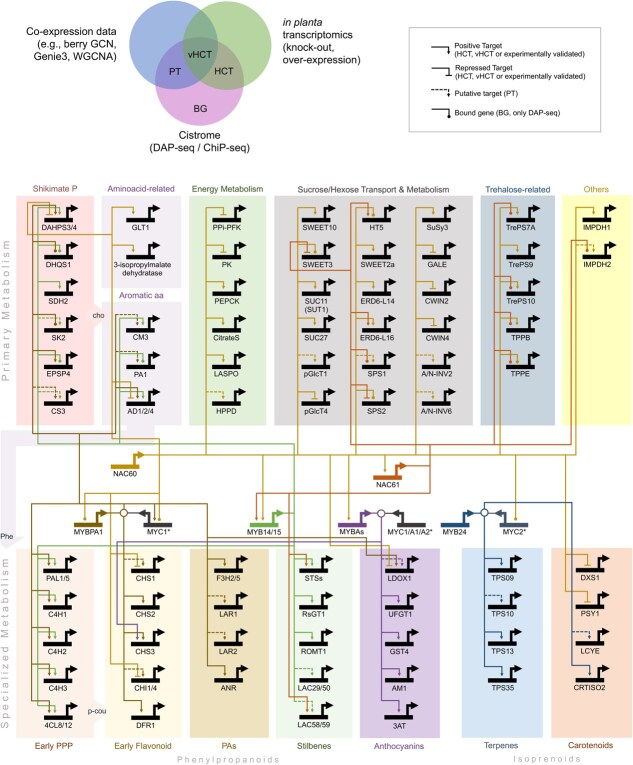
Integrative network-based framework for defining NAC-centered gene regulatory networks controlling grape berry ripening. (A) Conceptual workflow illustrating the integrative strategy used to classify TF targets. Target confidence levels are defined by the intersection of three independent data layers: TF–DNA binding evidence from cistrome analyses (DAP-seq and/or ChIP-seq), gene coexpression relationships derived from berry-focused GCNs (including WGCNA, GENIE3, and related approaches), and *in planta* transcriptomic responses obtained from transcription factor perturbation experiments (e.g. transient overexpression, stable overexpression, or knockout lines). Genes supported by multiple independent datasets are progressively assigned higher confidence levels, as illustrated by the Venn diagram. BG, bound genes; PT, putative targets; HCT, high-confidence targets; vHCT, very high-confidence targets. (B) Integrated NAC-centered gene regulatory network reconstructed from the reexploration of DAP-seq and transcriptional datasets of NAC60 and NAC61, highlighting both previously validated and newly identified regulatory targets. The network summarizes regulatory relationships between NAC60 and NAC61, downstream transcriptional regulators, and metabolic genes involved in primary metabolism (including shikimate and amino acid metabolism, energy metabolism, sucrose/hexose transport and metabolism, and trehalose-related pathways) and specialized metabolism (including phenylpropanoids, flavonoids, proanthocyanidins, stilbenes, anthocyanins, terpenes, and carotenoids). Target genes are classified as positively or negatively regulated, bound-only genes (DAP-seq), or putative targets according to the evidence supporting each interaction. NAC60 binding data considers any of the three DAP-seq analyses performed by D’Incà *et al*. [32], which either considered leaf-500 ng, leaf-1000 ng or berry-500 ng libraries. BG nodes represent bound-only candidates and are shown for completeness/hypothesis generation, but are not treated as HCT/vHCT unless supported by additional coexpression and/or perturbation transcriptomics layers. This integrative reanalysis expands the regulatory scope of NAC60 toward sugar transport, sugar signaling, and central metabolic pathways, and incorporates HCTs of additional TFs characterized through multiomics integration approaches discussed in this review. Gene symbols were taken from the Grape Gene Reference Catalogue and their corresponding identifiers are available in the Gene Cards application of the VitViz platform (https://plantaeviz.tomsbiolab.com/vitviz/catalogue/). MYBAs refer to MYBA1 and MYBA2. (*) MYC/bHLH factors are included due to their established interactions with MYB proteins in regulating numerous target genes.

Collectively, these studies evidence a combinatorial control and protein–protein interactions in NAC-centered GRNs positioning NAC TFs as central hierarchical regulators integrating developmental phase transitions with environmental and metabolic cues to control grape berry ripening, overripening, and stress-associated metabolic reprogramming. In this review, we further reinspected transcriptomic datasets from NAC-overexpressing grape plantlets and reintegrated them with DAP-seq binding landscapes to identify novel HCTs that were not initially reported in the original analyses describing these TFs. To facilitate functional interpretation and contextualization, all candidate target genes were systematically cross-referenced against the Grape Gene Reference Catalogue [54], the most comprehensive repository of curated grapevine gene names and functional annotations, enabling the identification of additional genes previously characterized in the literature. As summarized in Fig. 1, this integrative framework combines gene coexpression networks, DAP-seq data, and transcriptomic responses to transient TF overexpression to refine target selection and uncover additional regulatory layers. This expanded analysis revealed that NAC60 binds to and potentially regulates an extensive set of genes involved in primary metabolic pathways, including mono- and oligosaccharide biosynthesis, sugar transport, and sugar-related signaling pathways. Importantly, the resulting regulatory model incorporates not only NAC-centered HCTs but also the validated target genes of other TFs characterized through multiomics integration approaches that were discussed earlier in this review. Together, this synthesis represents the most comprehensive gene regulatory network of grape berry ripening assembled to date, providing a unified framework that connects developmental regulation, metabolic control, and environmental responsiveness.

Novel NAC60 targets include multiple members of the SWEET and Sucrose Transporter (SUT) families. SWEETs mediate sugar efflux and play central roles in apoplastic sugar partitioning during ripening [55–57], while SUTs mediate the active transport of sucrose across plasma membrane barriers in a process that is coupled to proton symport and that is critical for phloem loading [58]. Among these, *SWEET10* (bound at –2 and –1.5 kb from its TSS and induced with a logFC = 6 in NAC60-transiently overexpressing leaves) and *SUC11/SUT1* (bound at –3.7 kb and induced with a 1.79 logFC) emerge as clear candidate targets. Sucrose Synthase 3 (SuSy3)**,** previously linked to sucrose metabolism and carbon flux during berry development [59, 60], also arise as a potential NAC60-regulated gene. SuSy3 is bound at 24 bp from the TSS, is induced by NAC60 transient overexpression in leaves (1.5 logFC), and is highly correlated to NAC60 in berry-dependent GCNs. Also, the identification of several cell wall and vacuolar invertases, key enzymes catalyzing sucrose cleavage into glucose and fructose at veraison, is consistent with their established roles in driving hexose accumulation in ripening berries [56, 57]. The identification of multiple trehalose-6-phosphate synthases, such as *TrePS7a, TrePS9,* and *TrePS10*, and Trehalose-6-phosphate phosphatases (e.g. *TPPB*) further supports a role for NAC60 in sugar-related signaling pathways, given the central function of trehalose-6-phosphate as a metabolic signal integrating sugar status with developmental and hormonal cues. In conclusion, the identification of several of sugar-related enzymes among the newly proposed NAC60/61 target genes supports a model in which NAC TFs participate in the coordinated regulation of sucrose cleavage, hexose transport, and vacuolar storage during ripening. While this regulatory framework remains partially hypothetical and requires further experimental validation, it provides a mechanistic basis linking NAC-centered transcriptional control to the hallmark sugar accumulation that defines grape berry ripening.

### Integrating time-series transcriptome data to identify new roles of previously studied GRNs

The use of publicly available transcriptomic datasets capturing temporal and spatial gene expression dynamics can provide a powerful approach to uncover new roles for previously studied TFs, especially if combined with cistrome data. This approach enables the identification of previously uncharacterized regulatory interactions and the reassignment of novel functional roles to TFs in different biological contexts. For instance, TFs that were initially associated with a specific pathway may be shown to regulate additional genes under different environmental or tissue/developmental conditions. This integrative analysis also allows predicting the combinatorial actions of TFs and reveal spatiotemporal-specific regulatory modules.

**Figure 2:**
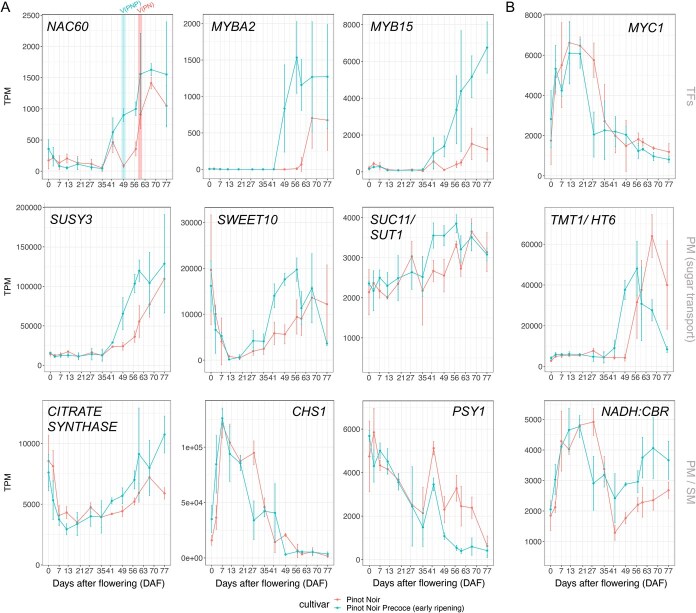
Integration of time-series transcriptomic data refines NAC60-regulated gene regulatory networks during grape berry ripening. (A) Reanalysis of publicly available time-series transcriptomic data from cv. ‘Pinot Noir’ (PN) and the early-ripening cv. ‘Pinot Noir Precoce’ (PNP) (Theine *et al*., 2021) confirming the previously proposed NAC60-centered GRN active at veraison. Genes shown in this panel correspond to established NAC60 HCTs and associated regulatory modules whose temporal expression patterns peak at ripening onset (veraison, ‘V’), and which show delayed expression in PN compared to PNP, supporting their involvement in veraison-associated metabolic and developmental transitions. (B) Expanded NAC60 regulatory network inferred from the same temporal transcriptomic dataset, highlighting novel candidate target genes corroborated by DAP-seq and whose expression profiles are temporally correlated with NAC60 activity but were not identified in earlier studies. These newly proposed targets are enriched in pathways related to sugar transport and accumulation, hormonal regulation, and specialized metabolism, indicating additional roles for NAC60 in coordinating metabolite production and ripening progression. Several of the targets shown here are proposed as negative regulatory targets of NAC60, including *PSY*, *CHS1*, and *MYC1*. Together, panels A and B illustrate how integrating time-resolved transcriptomic data with previously defined gene regulatory networks enables both validation of known regulatory interactions and the discovery of new context-dependent NAC60 functions during grape berry ripening (TFs: transcription factors; PM: primary metabolism; SM: specialized metabolism). Based on the 2014 and 2017 datasets reported by Theine *et al.* (2021), an online exploratory resource—the PN_vs_PNP expression atlas—was developed (https://plantaeviz.tomsbiolab.com/vitviz/PNvsPNP_atlas).

Here, we identified novel potential regulatory roles for *NAC60* in orchestrating ripening initiation and metabolite production thanks to the work of Theine *et al*. [61]. The authors investigated the genetic and molecular bases of ripening time variations in two closely related grapevine cultivars: cv. ‘Pinot Noir’ (PN) and the early ripening cv. ‘Pinot Noir Precoce’ (PNP). Performing transcriptomic analysis, the authors identified key differentially expressed genes (DEGs) across various developmental stages, focusing on their roles in ripening processes. They linked these temporal shifts to pathways involved in sugar metabolism, secondary metabolite production, and hormonal regulation, highlighting genes that may serve as candidates for controlling the timing of ripening, providing insights into berry development’s genetic determinism. This transcriptomic dataset resulted to be an invaluable resource for exploring the temporal dynamics of GRNs that control grapevine berry ripening. In fact, reanalyzing gene expression profiles at distinct developmental stages has enabled us to identify new NAC60 target genes involved in ripening-associated processes such as sugar accumulation, hormonal regulation, and specialized metabolite accumulation. In this way, we have confirmed the GRN previously proposed as regulated at veraison by NAC60 (Fig. 2A), while proposing new genes (Fig. 2B).

*HT6*/*TMT1* was identified among the new sugar transport-related genes associated with NAC-regulated networks. HT6 has been previously characterized as a plasma membrane hexose transporter whose expression increases sharply at veraison and correlates with the massive accumulation of glucose and fructose in ripening grape berries [62, 63]. After veraison, a substantial fraction of imported sucrose is cleaved into hexoses by cell wall invertases (CWINVs) and neutral invertases (NINVs), generating glucose and fructose that drive the rapid increase in berry sugar content. These hexoses are subsequently transported across the plasma membrane and into the vacuolar membrane by transporters such as HT6. Lu *et al*. [57] proposed that HT6 may contribute to sugar unloading and partitioning during ripening and that its activity is likely influenced by developmental and hormonal cues, including ABA. Notably, *HT6* is bound by NAC60 at ~2.8 kb upstream of the transcription start site, and although its expression is reduced upon NAC60 overexpression in leaves (−1.31 log-fold change; [32]), its developmental induction during ripening and delayed expression in cv. ‘Pinot Noir’ relative to cv. ‘Pinot Noir Precoce’ support its identification as a positive NAC60 target based on the PN/PNP time-series transcriptomic analyses. This apparent discrepancy likely reflects context dependence of NAC60 activity. NAC factors can function as activators or repressors depending on tissue, developmental stage, chromatin context, and availability of interacting cofactors [64]. Leaf overexpression assays may therefore capture NAC60-driven repression of *HT6* in a vegetative program, whereas in berry ripening NAC60 may participate in an activating complex and/or act indirectly by promoting upstream sugar/ABA programs that ultimately induce *HT6.* Importantly, this apparent discrepancy does not apply uniformly across all NAC60-bound targets.

### From grape NAC60 to strawberry RIF: conserved NAC control of nonclimacteric fruit ripening

In strawberry (Fragaria spp.), another nonclimacteric species, fruit ripening is defined by a well-characterized developmental transition from the white to the red stage, which marks the onset of ripening. This phase is characterized by rapid pigment accumulation, primarily anthocyanins, together with extensive cell wall remodeling leading to fruit softening and texture changes. Concurrently, metabolic reprogramming supports the accumulation of sugars and flavor-related compounds, contributing to overall fruit quality. Unlike climacteric fruits, these processes are largely governed by an ABA-driven regulatory program, which plays a central role in coordinating the transcriptional and physiological changes associated with ripening.

The RIPENING INDUCING FACTOR (RIF) has emerged as a master NAC regulator of strawberry fruit ripening. RIF is a close functional homolog of grape NAC60 and other related NACs found in grape [32]. In octoploid *Fragaria × ananassa*, stable FaRIF RNAi and overexpression lines demonstrated that RIF promotes coordinated ripening traits, including color formation, softening, and metabolic reprogramming, together with major shifts in central carbon metabolism during the white-to-red transition [65]. RIF acts upstream of ABA accumulation by inducing ABA biosynthetic genes such as *FaNCED3* and *FaZEP*, and RIF-silenced fruits in fact exhibited reduced ABA levels that can be partially rescued by exogenous ABA, supporting a positive feedback loop between ABA signaling and NAC activity. Transcriptomic analyses further identified RIF-dependent regulation of ABA- and light-responsive components, including *FaHVA22*, *FaSnRK2.6*, *FaHY5*, and *FaBBX19*, highlighting a central role for RIF in integrating hormonal and environmental cues during ripening [65, 66].

Additional studies in diploid *Fragaria vesca* showed that CRISPR–Cas9-mediated loss of *FvRIF* resulted in a near-complete block of fruit ripening, establishing RIF as an essential upstream regulator of the ripening program [34]. By integrating DAP-seq and RNA-seq, Li *et al*. [34] identified a large set of direct and indirect RIF targets enriched in pathways related to plant hormone signaling, starch and sucrose metabolism, and phenylpropanoid/flavonoid biosynthesis. These included core anthocyanin biosynthetic genes (*FvCHS*, *FvDFR*, *FvANS*, *FvUFGT*), cell wall-modifying enzymes (*FvPL2*, *FvPG2*, *FvEXP3*, *FvXTH*), and higher order transcriptional regulators such as the anthocyanin regulator *FvMYB10*, *FvSEP3*, *FvSPT*, and *FvARF2*, positioning RIF at the top of a hierarchical gene regulatory network coordinating pigmentation, softening, and developmental progression. Importantly, Li *et al*. [34] also revealed that RIF activity is post-translationally regulated through phosphorylation by FvMAPK6, with threonine 310 being critical for full transcriptional activity, thereby linking environmental signaling pathways directly to NAC-mediated ripening control. Complementary integrative analyses in cultivated strawberry, including ChIP-seq and TurboID-based proximity labeling, further expanded the RIF-centered regulatory landscape, supporting both direct and indirect regulation of key ripening processes, such as ABA biosynthesis and signaling, sugar accumulation and transport, flavonoid and lignin metabolism (e.g. *Fa4CL1*, *FaANR*, *FaC3H*, *FaPRX1*), cell wall remodeling, and aroma-related pathways, and identified additional NAC TFs that enhance RIF’s transcriptional activity through heterodimer formation [67].

**Figure 3:**
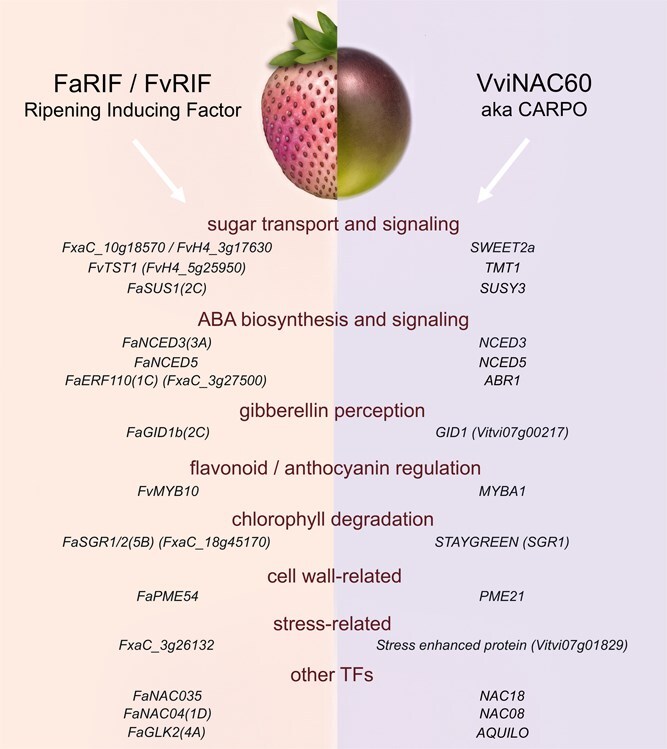
Conserved NAC-centered regulatory modules controlling nonclimacteric fruit ripening in grapevine and strawberry. Schematic representation of the comparative NAC-centered gene regulatory network integrating cistrome, transcriptomic, and orthology analyses in grapevine (*Vitis vinifera*) and strawberry (*Fragaria spp*.). The figure highlights some conserved regulatory roles of VviNAC60 in grapevine and its functional homolog RIF in strawberry. Genes shown represent shared orthologous NAC targets identified through the integration of DAP-seq (grapevine and *F. vesca*), ChIP-seq (cultivated strawberry), transcriptomics (found in [32, 34, 67]), and orthology inference using the AEGIS framework (Navarro-Payá *et al*., 2025). Conserved targets are grouped according to major biological processes associated with fruit ripening. The pathways highlighted (GA metabolism, cell wall remodeling, stress response, sugar transport and metabolism, ABA signaling, and chlorophyll/flavonoid transitions) were selected based on their consistent enrichment among RIF/NAC-bound targets identified through cistromic analyses. These modules represent core regulatory outputs repeatedly detected across independent datasets. Importantly, they correspond to measurable physiological hallmarks of ripening in both grape and strawberry, including hormonal shifts, softening, sugar accumulation, pigment transitions, and stress-associated reprogramming. Their inclusion therefore enables a cross-species comparison of conserved developmental modules underlying nonclimacteric fruit ripening. Species-specific regulatory branches are not shown, notably the stilbenoid biosynthetic pathway, which is under NAC-mediated control in grapevine but absent in strawberry. Gene IDs of grape and strawberry genes can be retrieved from the Gene Cards application of the StrawViz and VitViz modules within the PlantaeViz platform (https://plantaeviz.tomsbiolab.com/).

The recent integrative multiomics studies conducted in *Vitis* spp. and *Fragaria* spp. to delineate ripening gene regulatory networks demonstrate that these two species are emerging as robust plant models for dissecting the control of ripening initiation and progression in nonclimacteric fruits. With the aim of identifying conserved regulatory modules between grape and strawberry, i.e. modules that may also reflect shared mechanisms across other nonclimacteric fleshy fruits, we performed an orthology analysis between both species using the AEGIS framework [68]. This approach integrates multiple independent lines of evidence, including Pfam domain conservation, OrthoFinder clustering, MCScanX collinearity, and Best Reciprocal Blast hits (the resulting orthology relationships can be explored through the PlantaeViz platform [69]). Overall, we identified 6784 RIF-bound genes in strawberry cistrome datasets, corresponding to 4713 orthogroups, based on the integration of 14 135 peaks derived from both DAP-seq (*F. vesca*) and ChIP-seq (*F x* ananassa) experiments. When these data were compared with 11 329 NAC60-bound genes identified in grapevine, we detected an intersection of 2496 NAC-bound orthologous gene pairs (i.e. targets shared between all species). By further intersecting these orthology relationships with transcriptomic and cistrome datasets generated in recent grape and strawberry studies, we identified a core set of shared NAC HCT genes. These conserved targets are significantly enriched in pathways central to fruit ripening, including ABA biosynthesis and signaling (e.g. 9-*cis*-epoxycarotenoid dioxygenase [NCED] isoforms and ABA-responsive TFs [ABFs]), sugar transport and accumulation, and flavonoid biosynthesis, among others (Fig. 3). Interestingly, a few additional *NAC* genes were found as targets. In contrast, some regulatory branches were species-specific, such as the stilbenoid biosynthetic pathway, which is under NAC-mediated control in grapevine but absent in *Fragaria* spp. This branch is prominent in grapevine but not present in cultivated strawberry; thus, the species-specific signal may reflect lineage-level pathway reduction and/or divergence in target repertoires such that NAC/RIF homologs do not engage stilbene-related gene sets in strawberry.

Taken together, the comparative analyses presented here, integrating orthology inference with experimentally validated HCT repertoires, support the existence of a conserved NAC-centered regulatory hub in grapevine that mirrors the hierarchical and multilayered control exerted by RIF in strawberry. Within the comparative framework of this review, these homologous NAC TFs exemplify how the integration of ABA signaling, sugar metabolism, and secondary metabolic pathways underpins nonclimacteric fruit ripening, reinforcing the concept of conserved NAC-driven gene regulatory networks across fleshy fruit species.

## Future perspectives

Recent advances in integrative multiomics and network-based analyses have substantially improved our understanding of the gene regulatory networks underlying grape berry ripening and the molecular basis of phenotypic diversity across cultivars and environmental conditions. By combining transcriptomics, cistrome profiling, and systems biology approaches, this body of work provides a rich resource for constructing predictive GRNs and identifying key transcriptional regulators that control ripening initiation, progression, and associated metabolic transitions. These insights are not only fundamental for elucidating the biology of nonclimacteric fruit ripening but also offer actionable targets for breeding and biotechnological strategies aimed at optimizing fruit quality, harvest timing, and resilience to climate variability.

The temporal resolution of transcriptomic datasets has proven particularly powerful, allowing the inference of sequential gene activation and repression events and revealing regulatory hierarchies that govern the ripening process. Such temporal frameworks enable the identification of early transition markers, master regulators, and downstream executors of ripening-associated programs, thereby refining our capacity to model developmental trajectories and predict phenotypic outcomes.

Despite these advances, several key regulators remain insufficiently characterized. Future studies should prioritize the functional dissection of additional switch genes and ripening transition biomarkers, including TFs such as bHLH075 and WRKY19, originally identified by Fasoli *et al*. [43], as well as VERAISON1 (VER1), an ethylene response factor (ERF) and the causal gene underlying the *Ver1* locus (Frenzke *et al*. [24]). High-resolution genotyping-by-sequencing identified *Ver1* as the most likely causal candidate associated with variation in veraison timing in *V. vinifera*. Notably, *VERAISON1* is bound by NAC60 in its downstream 3′region and by NAC61 in both its promoter and downstream regions, suggesting a direct regulatory connection that warrants deeper investigation to clarify how NAC and ERF pathways intersect to control ripening onset and progression. In addition, other berry-specific NAC TFs identified in the network-based studies of Palumbo *et al*. [42] and Massonnet *et al*. [49] should be systematically explored to complete the hierarchical landscape of NAC-mediated regulation. Because NAC61-associated modules are enriched in dehydration/oxidative/biotic stress functions, these GRNs provide concrete entry points for climate-resilience strategies. For example, editing *cis*-elements in key NAC targets (or fine-tuning NAC dimerization interfaces) could decouple stress protection from undesirable trade-offs on fruit quality, enabling improved performance under heat, drought, and altered pathogen pressure.

Looking forward, future research should prioritize the integration of multiple omics layers generated both *de novo* and from public resources. Coupled with advanced network inference and modeling, this integration will be essential to predict short-term berry responses to environmental perturbations associated with climate change. Systems biology approaches enable a shift from descriptive to predictive frameworks, allowing the simulation of regulatory responses and the identification of actionable regulatory targets for crop improvement. In addition, future studies should expand beyond TF-centered regulation to incorporate other regulatory molecules, such as microRNAs and long noncoding RNAs. Emerging evidence in several fruit crops indicates that these molecules contribute to fine-tuning hormone signaling, stress responses, and developmental transitions. Incorporating these additional regulatory layers into integrative GRN models will be crucial for achieving a comprehensive and mechanistic understanding of fruit ripening and for fully exploiting the potential of systems biology in crop science.
